# CASCADE_SCAN: mining signal transduction network from high-throughput data based on steepest descent method

**DOI:** 10.1186/1471-2105-12-164

**Published:** 2011-05-17

**Authors:** Kai Wang, Fuyan Hu, Kejia Xu, Hua Cheng, Meng Jiang, Ruili Feng, Jing Li, Tieqiao Wen

**Affiliations:** 1 Laboratory of Molecular Neurobiology, School of Life Sciences and Institute of Systems Biology, Shanghai University, Shanghai 200444, China; 2 Department of Mathematics, College of Sciences, Shanghai University, Shanghai 200444, China

## Abstract

**Background:**

Signal transduction is an essential biological process involved in cell response to environment changes, by which extracellular signaling initiates intracellular signaling. Many computational methods have been generated in mining signal transduction networks with the increasing of high-throughput genomic and proteomic data. However, more effective means are still needed to understand the complex mechanisms of signaling pathways.

**Results:**

We propose a new approach, namely CASCADE_SCAN, for mining signal transduction networks from high-throughput data based on the steepest descent method using indirect protein-protein interactions (PPIs). This method is useful for actual biological application since the given proteins utilized are no longer confined to membrane receptors or transcription factors as in existing methods. The precision and recall values of CASCADE_SCAN are comparable with those of other existing methods. Moreover, functional enrichment analysis of the network components supported the reliability of the results.

**Conclusions:**

CASCADE_SCAN is a more suitable method than existing methods for detecting underlying signaling pathways where the membrane receptors or transcription factors are unknown, providing significant insight into the mechanism of cellular signaling in growth, development and cancer. A new tool based on this method is freely available at http://www.genomescience.com.cn/CASCADE_SCAN/.

## Background

Signal transduction plays an essential role in cell response to environment changes. This biological process is usually characterized by phosphorylation/dephosphorylation of some key proteins (e.g. kinases) and generally involves a signal cascade. The signal transduction process often starts from a membrane protein (usually a membrane surface receptor), spans a series of intercellular signaling proteins and then transfers to transcription factors in the nucleus, subsequently raising the expression of downstream genes. Studies demonstrate that many important cellular processes such as cell proliferation, differentiation, cell cycle control and cellular responses to nutrient limiting conditions are involved in different signaling pathways [1,2]. For example, Yokoi *et al *[3] demonstrated that hyperglycemia mediates endothelial cell senescence through the ASK1 signaling pathway. Tang *et al *[4] showed that the receptor kinase BRI1 and BR-signaling kinases (BSKs) mediate growth regulation related signal transduction in *Arabidopsis*. The Toll-like receptor (TLR) signaling cascade plays an essential role in recognizing and eliciting responses upon invasion of pathogens [5]. Recent high-throughput genomic and proteomic techniques, such as large-scale yeast two-hybrid (Y2H) [6], Co-Immunoprecipitation (Co-IP) [7,8], tandem affinity purification-mass spectrometry (TAP-MASS) [9,10], protein chip [11-14] and microarray experiments [15,16] have generated enormous amounts of data for uncovering signal transduction networks. This abundance of information brings increasing complexity to network analysis, which is a major obstacle to understanding the mechanisms of cell signaling.

Recently, computational methods have been introduced in mining signal transduction network. Steffen *et al *[17] developed a static model, NetSearch, to reconstruct the signal transduction network from PPI and gene expression data. For a given membrane protein and transcription factor, NetSearch will search for all possible linear paths that link the two proteins. By employing a depth first search (DFS) algorithm [17-20], paths of a specified length are kept, and then a statistical score is assigned to each path. Top scoring pathways are then assembled into the final branched signal transduction network. Liu *et al *[21] have worked on determining the order of signal transduction network components. They calculated the correlations between each gene pair and recorded the significance using a hypergeometric test to specify the correlation threshold. A score function is constructed to determine the final signal transduction network. Zhao *et al *[18,22] proposed a novel computational approach aimed at finding an optimal signal transduction network using an integer linear programming (ILP) and mixed integer linear programming (MILP) model. Similar approaches have also been proposed in more recent studies [20,23]. All those existing methods mainly use integrated PPI and gene expression data, which have been widely adopted in many related studies. They all aim at finding an optimal signal transduction network starting from a given membrane receptor and ending at a specific transcription factor. However, in most situations, we even do not know which membrane receptor or transcription factor is involved in a certain signaling pathway. In fact, most intermediate proteins are more easily available for their dominant position in quantity, which is neither a membrane receptor or transcription factor. These proteins could also be used in mining signal transduction networks. Besides, the datasets utilized in these methods are primarily based on experiments. Though the interactions are more reliable compared with computationally predicted interactions, the data is deficient. Some computational methods, e.g. gene co-expression [24] and semantic similarity of Gene Ontology (GO) annotations [25], indicate that genes with high scored interactions may be involved in the same signaling pathway [21]. However, this information either is limited or has not been incorporated in most databases constructed from experimental data. Though these interactions may not necessarily be direct interactions, using this information may help to improve prediction of signal transduction networks. We define "direct interaction" as a direct physical association between two proteins and "indirect interaction" as no direct physical association between two proteins in the actual state. Two proteins with indirect interaction must function through at least one medial protein.

**Figure 1:**
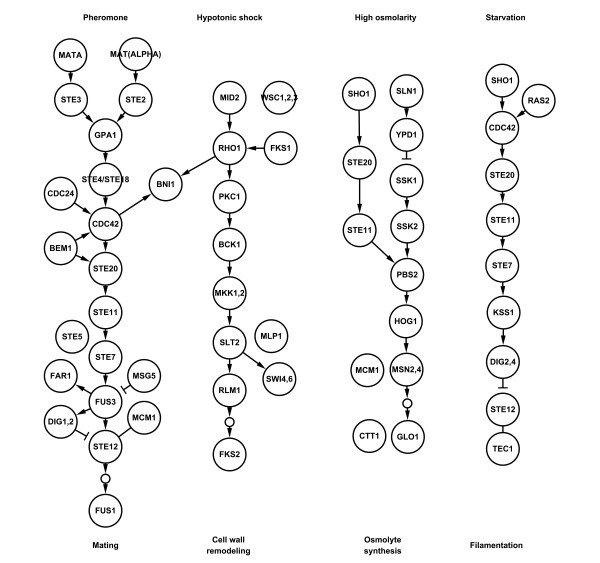
**The MAPK signaling pathways in yeast**. The data were obtained from the KEGG database.

Here, we present a novel computational method, named CASCADE_SCAN, under Linux system, to detect signal transduction networks from high-throughput data based on a customized steepest descent method (SDM) [26]. We do not mean to find a signal transduction network starting and ending from two specific proteins, but to find a high scored network within the top largest densities that contain a series of given proteins, also referred to as seed proteins, which are the supposed known components of a specific signaling pathway. These given proteins may also include the membrane receptor and the transcription factor. In addition, by searching for additional high related proteins automatically, indirect interactions are used effectively. This pre-processing is demonstrated below to be very useful. The well studied yeast MAPK signaling pathways (Figure 1), which have been widely used in previous studies, were also employed here to test our model. All figures were generated by Cytoscape [27]. The results indicate that the precision and recall values are comparable with those of other existing methods, even though the dataset we used is much larger than those previously utilized.

## Results

Since the yeast MAPK pathways have been well studied and widely used as reference standards in previous studies [17,18,21,23], we thus used those pathways (Figure 1) downloaded from the KEGG database [28] to validate the reliability of CASCADE_SCAN. In the following steps, we discuss the results of the pheromone response and the filamentous growth pathway, respectively. It is worth noting that the seed proteins we used were not restricted to membrane receptors and transcription factors, as in previous studies.

### Detecting the pheromone response pathway

**Figure 2:**
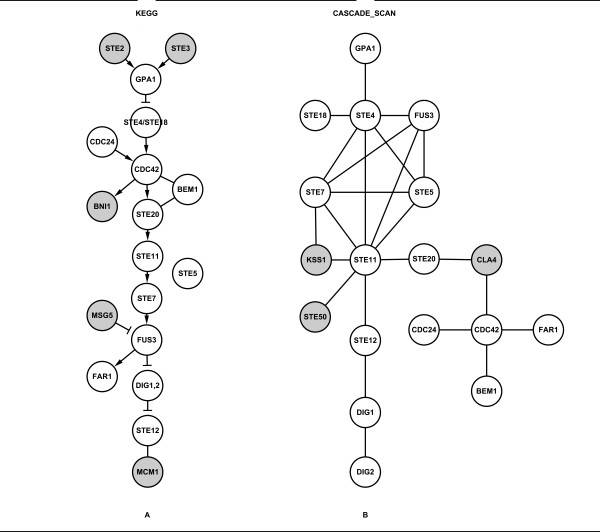
**The pheromone response pathway**. (A) The pheromone response pathway obtained from KEGG. The darker nodes indicate nodes in the KEGG pheromone response pathway but not detected by CASCADE_SCAN. (B) The CASCADE_SCAN output for the pheromone response pathway (74% recall and 82% precision). The darker nodes indicate detected by CASCADE_SCAN but not in the KEGG pheromone response pathway. (PPI score threshold: 0.800, credible PPI score threshold: 0.980, DFS path length: 2, seed proteins: CDC24, DIG1, FAR1 and STE4).

The pheromone response pathway (Figure 2) mediates cell signaling in response to extracellular peptide pheromones. In the current KEGG database, there are about twenty proteins present in the pheromone response pathway, as shown in Figure 2A. We randomly selected four seed proteins and varied the score threshold from 0.800 to 0.950 with an interval of 0.050. Different parameters were tested (Additional file 1). Table 1 shows the results of the performance evaluation mentioned above compared with other methods (color-coding, NetSearch, PathFinder, ILP). We can see that CASCADE_SCAN obtains ~76% recall after 20 independent experiments, which is comparable with color-coding, NetSearch and PathFinder, though the ~54% precision is lower (Table 1 and Additional file 2). The p-values detected by both the hypergeometric test and Fisher's exact test from DAVID are less than or equal to 2.43E-16 (Table 2), indicating the effectiveness of our method.

**Table 1:** Performance comparison between different methods in precision and recall for pheromone response pathway.

Method	Precision (%)	Recall (%)
CASCADE_SCAN	54	76
color-coding	83	75
NetSearch	74	70
PathFinder	88	75
ILP (λ = 0.50)	47	80

The results of CASCADE_SCAN were obtained from 20 independent experiments using four seed proteins (PPI score threshold: 0.800, credible PPI score threshold: 0.980, DFS path length: 2). Note, the results of color-coding algorithm, NetSearch, PathFinder and ILP were obtained from Zhao's paper.

**Table 2:** P-values of functional enrichment for pheromone response pathway.

GO Term	Proteins annotated by DAVID in our method (total: 18)	Proteins annotated by DAVID in SGD (total: 4870)	P-value (hypergeometric test)	P-value (DAVID Fisher test)
GO:0000750~pheromone-dependent signal transduction involved in conjugation with cellular fusion	13	29	4.18E-27	8.61E-25
GO:0032005~signal transduction involved in conjugation with cellular fusion	13	29	4.18E-27	8.61E-25
GO:0019236~response to pheromone	16	105	9.89E-26	4.74E-24
GO:0031137~regulation of conjugation with cellular fusion	13	36	1.41E-25	2.06E-23
GO:0046999~regulation of conjugation	13	36	1.41E-25	2.06E-23
GO:0000749~response to pheromone during conjugation with cellular fusion	14	69	9.51E-24	6.41E-22
GO:0000747~conjugation with cellular fusion	15	119	2.06E-22	7.92E-21
GO:0000746~conjugation	15	125	4.49E-22	1.64E-20
GO:0019953~sexual reproduction	16	277	nan	1.78E-17
GO:0048610~reproductive cellular process	15	243	nan	2.43E-16

Note: "nan" is beyond the computer's capability.

Figure 2B shows the results detected by CASCADE_SCAN. Though our dataset included many computationally predicted interactions, the precision and recall reached a high level. It can be seen that 15 proteins in the KEGG pheromone response signaling pathway are detected by our method except for STE2, STE3, BNI1, MSG5 and MCM1 (Figure 2A and 2B). However, several additional proteins, KSS1, STE50 and CLA4, are also detected by our method (Figure 2B).

We note, however, that some of those proteins not in the KEGG pheromone response signaling pathway were also detected by some of the other methods (color-coding, NetSearch, PathFinder, ILP). For example, KSS1 (mitogen-activated protein kinase) was also presented in the pheromone response pathway by our method (Figure 2B). In a previous study, KSS1, together with FUS3, was demonstrated to regulate the signal transduction cascade of the pheromone response and filamentous growth pathways [29]. KSS1 also activates the transcription factor STE12 as well as phosphorylates DIG1, DIG2 and STE12, which are involved in both the pheromone response and filamentous growth pathways [30,31]. Moreover, KSS1 was also detected by all of the other four methods. CLA4 is a member of the STE20 subfamily, which belongs to the STE Ser/Thr protein kinase family. CLA4 may play a role in the phosphorylation of alpha-factor-arrested yeast [32]. STE50 was reported to interact with the MAPKKK STE11 through the respective SAM domain [33,34], and is required in growth arrest during conjugation at an early step in yeast mating [35]. We, therefore, can draw the conclusion that those proteins indeed have some association with the yeast pheromone response pathway, though they are not included in the KEGG pheromone response pathway.

BNI1, which was not detected by most of the other methods (color-coding, NetSearch, PathFinder, ILP), was also not detected by CASCADE_SCAN. This may be because it has both weaker and fewer interactions with other proteins in this pathway, which may also have been the case for the other proteins STE2, STE3, MSG5 and MCM1.

### Detecting the filamentous growth pathway

**Figure 3:**
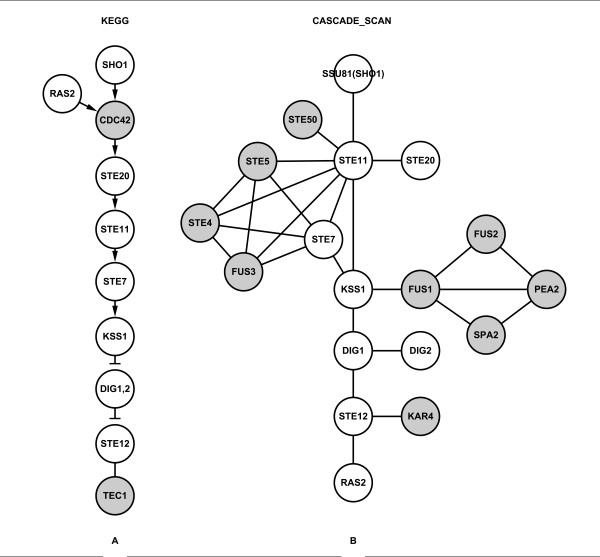
**Filamentous growth pathway**. (A) The filamentous growth pathway obtained from the KEGG database. The darker nodes indicate nodes in the KEGG filamentous growth pathway but not detected by CASCADE_SCAN. (B) The CASCADE_SCAN output for the filamentous growth pathway (82% recall and 50% precision). The darker nodes indicate nodes detected by CASCADE_SCAN but not in the KEGG filamentous growth pathway. (PPI score threshold: 0.900, credible PPI score threshold: 0.950, DFS path length: 5, seed proteins: KSS1, RAS2 and SSU81).

The filamentous growth pathway (Figure 3) regulates cellular response to nutrient limiting conditions. For this pathway (Figure 3A), there are many common proteins with other MAPK pathways (Figure 1). So, we randomly selected three or four seed proteins only from SSU81(SHO1), TEC1, RAS2 and KSS1. Different parameters were also tested (Additional file 3). After five independent experiments, we obtained an average of ~89% recall and ~36% precision (Table 3 and Additional file 4). Table 3 shows the performance of CASCADE_SCAN in detecting the filamentous growth pathway compared with that of NetSearch, PathFinder and ILP. CASCADE_SCAN clearly shows both higher recall and precision than all the other methods. In addition, the p-values detected by both the hypergeometric test and Fisher's exact test from DAVID are less than or equal to 4.42E-23 (Table 4), indicating that our method is effective.

**Table 3:** Performance comparison between different methods in precision and recall for filamentous growth pathway.

Method	Precision (%)	Recall (%)
CASCADE_SCAN	36	89
NetSearch	33	64
PathFinder	28	82
ILP (λ = 0.50)	29	73

The results CASCADE_SCAN were obtained from four independent experiments using three seed proteins, and one experiment for four seed proteins. (PPI score threshold: 0.900, credible PPI score threshold: 0.950, DFS path length: 5). Note, the results of color-coding algorithm, NetSearch, PathFinder and ILP were obtained from Zhao's paper.

**Table 4:** P-values of functional enrichment for filamentous growth pathway.

GO Term	Proteins annotated by DAVID in our method (total: 17)	Proteins annotated by DAVID in SGD (total: 4870)	P-value (hypergeometric test)	P-value (DAVID Fisher test)
GO:0030447~filamentous growth	16	113	3.96E-26	1.84E-24
GO:0040007~growth	16	138	1.19E-24	4.42E-23

Figure 3B shows the output of CASCADE_SCAN when using SSU81, RAS2 and KSS1 as seed proteins. CASCADE_SCAN identified a total of 18 proteins (Figure 3B). The 11 proteins in the KEGG filamentous growth pathway are shown in Figure 3A, nine of which were detected by CASCADE_SCAN except for CDC42 and TEC1 (Figure 3B). We investigated the nine additional proteins, and found most of them were identified in the pheromone response pathway, including STE4, STE5, FUS3 and FUS1. This sharing proteins phenomenon was also observed in previous studies [18,23]. In addition, PEA2, SPA2, KAR4, STE50 and FUS2, which are not mentioned in any KEGG MAKP pathway, were newly detected by our method (Figure 3B). Those proteins were detected mainly because the integrated database contains computationally predicted PPIs with high scores and no protein activity data were utilized.

### Detecting the cell wall remodeling and high osmolarity pathways

**Figure 4:**
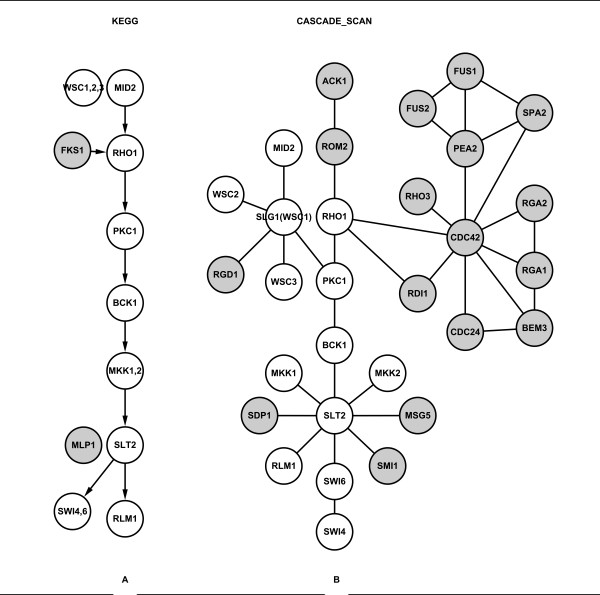
**Cell wall remodeling pathway**. (A) The cell wall remodeling pathway obtained from the KEGG database. The darker nodes indicate nodes in the KEGG cell wall remodeling pathway but not detected by CASCADE_SCAN. (B) The CASCADE_SCAN output for the cell wall remodeling pathway (87% recall and 43% precision). The darker nodes indicate nodes detected by CASCADE_SCAN but not in the KEGG cell wall remodeling pathway. (PPI score threshold: 0.800, credible PPI score threshold: 0.980, DFS path length: 5, network size threshold: 30, seed proteins: MKK1, SLG1 and WSC2).

**Figure 5:**
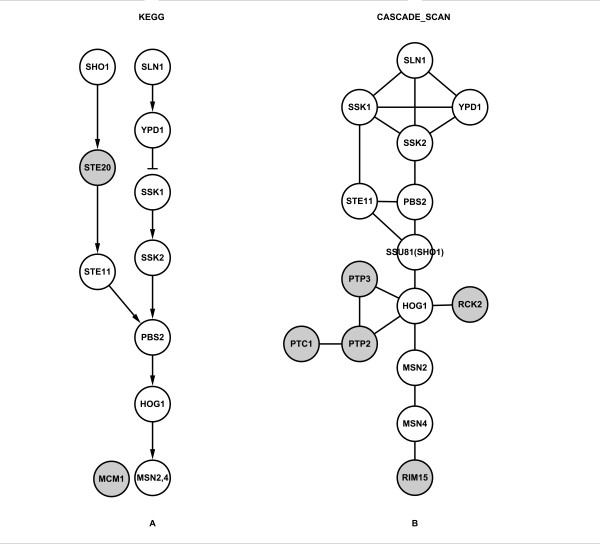
**Osmolyte synthesis pathway**. (A) The osmolyte synthesis pathway obtained from the KEGG database. The darker nodes indicate nodes in the KEGG osmolyte synthesis pathway but not detected by CASCADE_SCAN. (B) The CASCADE_SCAN output for the osmolyte synthesis pathway (83% recall and 67% precision). The darker nodes indicate nodes detected by CASCADE_SCAN but not in the KEGG osmolyte synthesis pathway. (PPI score threshold: 0.800, credible PPI score threshold: 0.980, DFS path length: 5, network size threshold: 30, seed proteins: MSN2, PBS2 and SLN1).

We also evaluated the cell wall remodeling (Figure 4) and high osmolarity (Figure 5) MAPK pathways. These two KEGG pathways are shown in Figure 4A and 5A. After 20 independent experiments by randomly selected three seed proteins each time, CASCADE_SCAN obtained ~69% recall and ~21% precision for the cell wall remodeling pathway (Table 5 and Additional files 5 and 6), and ~77% recall and ~20% precision for the high osmolarity pathway (Additional files 7 and 8), using a default network size threshold of 50. Though the recall values are relatively high (Table 5), the precision seems to be very low. Generally speaking, the more complex the organism is, the more components a signaling pathway contains. Through our investigation of all the KEGG signaling pathways, the number of signaling pathway components usually does not exceed 50 in higher organisms (e.g. human), and 30 in relatively lower organisms (e.g. yeast and fly). Therefore, we also evaluated the two pathways with a network size threshold of 30. CASCADE_SCAN obtained ~64% recall and ~32% precision for the cell wall remodeling pathway (Table 5), and ~82% recall and ~39% precision for the high osmolarity pathway, showing significant improvement in the precision at a relative high level. Moreover, all of the p-values are less than or equal to 8.58E-17 (Table 6) and 5.85E-11 (Table 7), respectively, indicating that our method is effective.

**Table 5:** Performance comparison between different methods in precision and recall for cell wall remodeling pathway.

Method	Precision (%)	Recall (%)
CASCADE_SCAN (s = 50)	21	69
CASCADE_SCAN (s = 30)	32	64
ILP (λ = 0.15)	56	63
NetSearch	50	56

The results CASCADE_SCAN were obtained from 20 independent experiments using three seed proteins. (PPI score threshold: 0.800, credible PPI score threshold: 0.980, DFS path length: 5). Note, the results of NetSearch and ILP were obtained from Zhao's paper.

**Table 6:** P-values of functional enrichment for cell wall remodeling pathway.

GO Term	Proteins annotated by DAVID in our method (total: 26)	Proteins annotated by DAVID in SGD (total: 4595)	P-value (hypergeometric test)	P-value (DAVID Fisher test)
GO:0030427~site of polarized growth	22	235	nan	3.34E-24
GO:0044463~cell projection part	15	113	1.72E-18	4.57E-17
GO:0042995~cell projection	15	118	3.39E-18	8.58E-17

Note: "nan" is beyond the computer's capability.

**Table 7:** P-values of functional enrichment for osmolyte synthesis pathway.

GO Term	Proteins annotated by DAVID in our method (total: 14)	Proteins annotated by DAVID in SGD (total: 4870)	P-value (hypergeometric test)	P-value (DAVID Fisher test)
GO:0006970~response to osmotic stress	13	104	1.23E-21	6.01E-20
GO:0009628~response to abiotic stimulus	14	344	nan	8.80E-16
GO:0033554~cellular response to stress	13	562	nan	5.85E-11

Note: "nan" is beyond the computer's capability.

Figures 4B and 5B show the results of the cell wall remodeling and the high osmolarity pathways detected by CASCADE_SCAN. As we can see in the figures, most components of the two pathways are detected by CASCADE_SCAN. However, the integrated database contains many more interactions, so several other proteins were also detected. In addition, CASCADE_SCAN seems to predict fewer edges between the proteins in the predicted signal transduction networks comparing with other methods. Hence, even though the membrane receptor and transcription factor are not known, we still know where the signal is from and to among those proteins, since most proteins have only one link to the preceding and succeeding element in the predicted network. In fact, if we require the order between the proteins to be more intuitive, fewer edges should be kept in the predicted network. We achieved this goal by maximizing the average weight of the network while keeping most of the reliable interactions, as described by equation (3).

## Discussion

Generally, some potential proteins involved in a signaling pathway stimulated by environmental factors are easily available through various reliable means, such as manual literature curation and biological experiments. But in most situations, not all or none of these proteins are membrane receptors or transcription factors. Moreover, the proteins we obtained may be more than just two proteins (a membrane receptor and a transcription factor). Therefore, CASCADE_SCAN is more suitable for actual biological application compared with existing methods such as color-coding, NetSearch, PathFinder and ILP. Nevertheless, although those methods utilize a more reliable dataset, the data is limited. However, using computationally predicted interactions may make up for the deficiency of experiment data, which is also one of our original aims.

In addition, we used a different but both a reasonable and effective data pre-processing scheme for CASCADE_SCAN. Firstly, we used a customized DFS algorithm to search for common nodes within a certain path length. We found that, in the integrated database (incorporating the Search Tool for the Retrieval of Interacting Genes/Proteins (STRING) and Biological General Repository for Interaction Datasets (BioGRID) databases), more than 90% nodes can link with any other nodes within a six-path length, and proteins in the same pathway often within a two-path length, which is different from the previously reported 6-9 steps. This is primarily because the integrated database stores many more indirect PPIs that were primarily generated by computational methods. The indirect interaction weights are often lower than the direct interaction weights but much higher than the weights between two unrelated nodes. Therefore, the path length between any two nodes would become correspondingly shorter, and we used a relatively shorter path length for the DFS algorithm here. Secondly, proteins involved in the same signaling pathway usually have a similar gene expression profile, so there are usually more and stronger interactions among them than others in the integrated database. And vice versa, many computationally predicted and high scored PPIs also indicate that the corresponding proteins may be involved in the same signaling pathway. Our approach--searching for additional seed proteins--was based on this hypothesis. When detecting the pheromone response pathway, by providing only four seed proteins, CDC24, DIG1, FAR1 and STE4, seven other proteins were detected in the pre-processing step (PPI score threshold: 0.800, credible PPI score threshold: 0.980, DFS path length: 2), including CDC42, BEM1, STE12, STE11, FUS3, STE18 and STE5, all of which are in the KEGG pheromone response pathway. When determining the filamentous growth pathway, by only giving three seed proteins KSS1, RAS2 and SSU81, three other proteins including FUS1, STE12 and STE11 were detected in the pre-processing step (PPI score threshold: 0.900, credible PPI score threshold: 0.950, DFS path length: 5), of which STE12 and STE11 are in the KEGG filamentous growth pathway except for FUS1. In all, these pre-processing schemes were demonstrated to be effective in inferring signaling pathways when using the integrated database.

Recent studies have indicated that genes involved in the same signaling pathway tend to have similar gene expression profiles, which is especially notable regarding adjacent pathway components [21]. Moreover, signal transduction usually shows different activation under different situations [18]. Based on those hypotheses, microarray expression data [36,37] were employed in previous studies as a complement to PPI data. Recently, studies have reported that using fold change criterions in the pre-processing schemes removes genes without significant change [18]. However, this process may also eliminate some important genes without significant quantity change in mRNA level, but showing activity changes in protein level. Therefore, fold change was not incorporated in this study. However, fold change criterion of gene expression data could be easily incorporated into the filtering process. Besides, an appropriately bigger score threshold, a relatively shorter DFS path length and a more stricter PPI scoring system would be helpful for excluding most irrelevant proteins as well as most indirect interactions.

When we attempted to compare the results of color-coding, NetSearch, PathFinder and ILP obtained from Zhao's paper [18], we found the results are actually incomparable because we used a different dataset and method. Firstly, the integrated dataset we used contained 5,717 yeast proteins and 322,084 yeast protein interactions, much larger than the previously used dataset. Moreover, most of these interactions are indirect interactions, while the previously used dataset contains mostly direct interactions. Secondly, the seed proteins used in our method are not confined to membrane receptors or transcription factors, and are usually more than just two proteins. Using different seed proteins may also lead to different precision and recall values. Therefore, it is difficult to say which method is better. Furthermore, the PPI scoring system is of the most importance, for which we combined the topological clustering semantic similarity (TCSS) scoring and STRING scoring systems. The TCSS scoring system predicts PPI from semantic similarity based on GO annotations [38]. The STRING scoring system method predicts PPI from various methods, such as the neighbourhood method, fusion events, co-occurrence, co-expression, experimental methods and text-mining. The combined scoring system seems to be stricter, and may be one of the reasons for our better performance. Nevertheless, we still recommend using other methods as complements for predicting signal transduction networks because no single method is perfect. More work needs to be done in this field as well as controlling for network size.

We note that the precision values are much lower for the other three pathways (filamentous growth, cell wall remodelling, and high osmolarity pathways) compared to the pheromone response pathway. This is primarily because most proteins in each of the other three pathways also have strong interactions with other proteins that are in other pathways. Therefore, more proteins are predicted than for the other three pathways. For instance, CDC42, STE20, STE11, STE7, DIG1, DIG2 and STE12 in the filamentous growth pathway could interact with BEM1, PBS2, FUS3, MCM1, STE4, STE5, STE18 and CDC24 in the pheromone response pathway, STE11 could interact with PBS2 in the high osmolyte pathway, and RAS2 could interact with CYR1 in the meiosis pathway. This is the sharing proteins phenomenon as mentioned in previous studies [18,23]. Though all the methods predicted mostly true proteins, i.e., with high recall values, the other three pathways contain fewer proteins than the pheromone response pathway. Thus, the precision for determining these signal transduction networks is relatively lower. Another reason may be there is less sufficient PPI information available for the other three pathways compared with the well studied pheromone response pathway.

## Conclusions

In this paper, we reported a new method, named CASCADE_SCAN, to detect signal transduction networks from high-throughput data. A new tool based on this method is freely available from the website http://www.genomescience.com.cn/CASCADE_SCAN/. Different from previous methods, in CASCADE_SCAN, the SDM is employed for inferring the signal transduction network and the given proteins utilized are not confined to membrane receptors or transcription factors. We also demonstrated that indirect interactions (e.g. the most computationally predicted interactions) can be effectively used in mining signaling transduction networks. This is particularly useful because all databases will include more and more indirect interactions as the data accumulates.

## Methods

### Data preparation

Widely used datasets often include PPI data and gene expression data. Here, only the PPI dataset was employed. The Yeast Proteome Database (YPD) [39,40], Saccharomyces Genome Database (SGD) [41] and Database of Interacting Proteins (DIP) [42,43] are the most frequently used PPI databases, but the interaction dataset in those databases is very limited, which may lead to misconnections due to deficient data. In this study, we constructed a PPI dataset from the STRING database (Version 8.3) [24,44] and BioGRID database (Version 3.1.74) [45]. The current STRING database contains 6,015 yeast proteins and 245,782 yeast protein interactions, and the BioGRID database contains 6,063 yeast proteins and 168,599 yeast protein interactions. Our combined database contains both direct and indirect PPIs derived from both computational methods and biological experiments, providing more comprehensive information than previously used.

### PPI scoring system

To score the PPI pairs in the combined database, we integrated the STRING scoring system and the TCSS method [25]. The STRING database infers PPIs through various approaches, including the neighbourhood method, fusion events, co-occurrence, co-expression, experimental methods and text-mining. It integrates all probabilities of those methods and assigns each PPI pair a reasonable score [24]. The original PPI score in STRING database is from 0 to 999, which is subsequently normalized from 0.000 to 0.999 by dividing by 1000. For all the PPI pairs in the combined database, the TCSS algorithm was implemented to give each PPI pair a score with a default biological processes cutoff of 3.5. The score is normalized from 0.000 to 0.999 by multiplying by 0.999. However, not all of the proteins would have a corresponding GO term in the annotation file. PPI pairs having neither a score in the STRING database nor a TCSS score were removed. Finally, we obtained 5,717 yeast proteins and 322,084 yeast protein interactions. The combined score (the weight of an edge) of each PPI pair is calculated as:(1)

where, *S_TCSS _*is the normalized score calculated by TCSS and *S_STRING _*is the normalized STRING score. Note, if there is a PPI pair only in the STRING database or could not be scored by TCSS, *S_TCSS _*would be 0, and if the PPI pair is not presented in the STRING database, *S_STRING _*would be 0.

### Data pre-processing

To reduce the false positive rate, two pre-processing steps were carried out to exclude obviously irrelevant proteins but to keep the high correlated proteins as much as possible.

Firstly, given several seed proteins, which we assumed to be known components in a signaling pathway, a similar but different DFS algorithm was realized. Our DFS algorithm would search for all proteins connected with each seed protein within a certain path length. The common proteins within this scope were kept.

In addition, given an interaction score threshold and several seed proteins, CASCADE_SCAN searches for additional proteins having common association with the seed proteins. If a protein (the nodes in the network graph) possesses at least three interactions (the edges in the network graph) with the given seed proteins, this protein would be selected as a new seed protein, which would also be considered as a component of the signal transduction network. Besides, a protein, with at least two high credible interactions with the given seed proteins, would be also selected as a new seed protein. All of the seed proteins would be included in the calculation of the distance or score when we selected the steepest descent nodes or edges, as described in the following steps.

These steps were carried out by CASCADE_SCAN automatically. The parameters are adjustable. By default, the path length is set as 10 and the credible edge weight threshold 0.980.

### Customized steepest descent method for detecting signaling transduction networks

Given PPI information, our model is based on the following hypotheses: Firstly, proteins in the same signaling pathway have stronger interactions than that in different signaling pathways. Secondly, proteins have more interactions with each other in the same signaling pathway than in different signaling pathways. Thus, nodes in the same network usually have shorter distances to each other than nodes in different networks, and an "actual network" usually processes a higher density than networks containing more unrelated nodes. The density of a network is calculated as shown in equation (2). However, there is still a problem: networks composed of parts of the "actual network" may also have a similar or even much higher density. To resolve this problem, we adopted a strategy to balance the network density and size. We selected the top five networks sorted by network density in descending order, and the network of the largest size from among the five as the most possible one containing the most components of the "actual network". Generally, the more and stronger the interactions, the higher the total score. Our goal was to find a compact network with the highest score. The score of a network is calculated as shown in equation (4).

To achieve our goal, the SDM, which is the simplest of the gradient methods, was employed to remove obviously irrelevant nodes. The basic idea of the method is choosing a direction where the objective function decreases most quickly. The optimization process starts at a certain point and slides along the direction until the result get close enough to the final solution. We detail this process below. Firstly, the interaction score between two proteins is converted to a distance. An interaction score below the score threshold is reassigned as 0, which denotes there is no interaction between the two proteins, and the corresponding distance will be considered as an infinite number. Otherwise, if the interaction score is above the score threshold, the corresponding distance will be assigned as 1 - score. Then, the graph search algorithm Dijkstra [46] is employed to calculate the distance between any two proteins. Given the initial node, this algorithm will find the shortest path between this node and any other node in the graph. Hence, it is often used in solving routing related problems. Consequently, the distances between each protein and any seed protein are obtained. Generally speaking, if the distance of a node to the "actual network" is maximal, the node represents the steepest descent direction. However, the "actual network" is unknown, we simply use the distance of the node to any of the given nodes (i.e. seed proteins) as the distance to the "actual network". Hence, the node corresponding to the maximal distance will be selected as a candidate steepest descent node to remove from the network. While, if removing the candidate steepest descent node leads to a disconnection between given nodes, the node corresponding to the next maximal distance is selected as a candidate steepest descent node, and so forth.

However, there still may be many nodes with short distances to some of the given nodes. So, when the network size is reduced to a certain extent, using the distances will no longer be able to distinguish the differences among those nodes. Moreover, proteins in the same pathway usually have more than one high scored interaction predicted by a computational method. Futhermore, the aggregate scores, which represent the contributions of a certain node to all of the given nodes, are obviously distinct from distances to distinguish which node should be selected as the candidate steepest descent node. For instance, suppose there are two nodes, N1 and N2, and three given nodes, G1, G2 and G3. The edge weight (interaction score for two proteins) between N1 and G1, N1 and G2, and N1 and G3 are 0.850, 0.900 and 0.95, respectively. While, there is no edge between N2 and G3, the edge weights between N2 and G1, and N2 and G2 are 0.950 and 0.900, respectively. Therefore, the distance between N1 and the given nodes (G3 corresponding to the maximal distance) is 0.050, and the distance between N2 and the given nodes (G1 corresponding to the maximal distance) is also 0.050. That is to say, there is no difference between N1 and N2 in distance. However, the aggregate contributing score of N1 (2.700) is much higher than the aggregate contributing score of N2 (1.850). So, when all of the remaining nodes have at least one edge with the given nodes, the aggregate scores are employed to select the candidate steepest descent node.

When the network size is below a specific threshold (by default, the maximal network size is restricted to 50), the top five largest density networks are recorded. However, these networks include many false negative edges, which represent mostly indirect interactions in the networks. To remove these false negative edges as much as possible, we use a maximal average weight controlling strategy, and the steepest descent method is employed here again. If an edge weight in the current network is minimal, removing this edge would maximize the average weight the most quickly. So, the least weighted edge is selected to be removed from the network until the average weight peaks. The average weight of a network is calculated as shown in equation (3). However, there may be more than one edge having the same weight, and all of them would be minimal. In this situation, we randomly select an edge from among them as the candidate steepest descent edge. If the removal of the edge leads to disconnection of the network components, the next least weighted edge is selected immediately. Finally, the aggregate score of each network is calculated, the network with the highest score being the most possible one. The formulas to illustrate the process are shown in detail below:(2)(3)(4)(5)

where, *i *is the *i*-th node, *j *is the *j*-th node and *n *is the total number of nodes in a signal transduction network, respectively, and each node in the signal transduction network represents a protein. *e_ij _*is the edge between node *i *and *j*, which represents whether the interaction between node *i *and *j *is selected as part of the network or not. If the edge between node *i *and *j *is selected, *e_ij _*is 1, otherwise, *e_ij _*is 0. *w_ij _*is the interaction weight of edge *e_ij_*, which is represented by the PPI score. We consider the network as an undirected graph here. Therefore, interaction weights *w_ij _*and *w_ij _*are the same in nature. *D_n _*is the density of a network with a specific size of *n*. *W_n _*is the average weight of a network with a specific size of *n*. Each time a least weighted edge is removed from the network, the average weight becomes larger. *S_n _*is the score of the network with a specific size of *n *when the average weight peaks.

To date, the network with the highest score has been obtained. For comprehensive consideration, we extended this restriction to the top N highest scored network.

### Performance evaluation

We employed precision and recall rates to evaluate the performance of our model. Precision is defined as the rate of the number of correctly predicted nodes to the total number of nodes determined in the signal transduction network. The recall rate is defined as the correct number of predicted proteins to the total number of proteins in the corresponding KEGG pathway. Statistical significance was also measured by functional enrichment analysis of the network components. These test methods are recognized as common evaluation criteria for signal transduction networks [17,18,23].

Different from previous repeat experiments, we randomly selected several proteins in the same pathways as seed proteins, rather than using the same membrane receptor and transcription factor as the seed proteins.

GO functional enrichment analysis was performed by DAVID [47,48]http://david.abcc.ncifcrf.gov/. We also employed hypergeometric distribution to calculate the statistical significance of the enriched network components, as described by:(6)

where *N *represents the total number of proteins in the SGD database, *n *represents the number of proteins with the specific function annotated by DAVID in *N *proteins, *M *denotes the network size and *x *is the number of proteins with the specific function annotated by DAVID in *M *proteins. Fisher's exact test p-values were also obtained from DAVID functional enrichment analysis.

## Authors' contributions

KW completed the study and wrote the manuscript. FH and KX checked the mathematical algorithm. HC, MJ, RF and JL checked the results. TW supervised the project. All authors read, revised and approved the final manuscript.

## Supplementary Material

Additional file 1 **The output of CASCADE_SCAN for detecting the pheromone response pathway using different parameters**. Click here for file

Additional file 2 **Seed proteins and the output of CASCADE_SCAN for detecting the pheromone response pathway**. Click here for file

Additional file 3 **The output of CASCADE_SCAN for detecting the filamentous growth pathway using different parameters**. Click here for file

Additional file 4 **Seed proteins and the output of CASCADE_SCAN for detecting the filamentous growth pathway**. Click here for file

Additional file 5 **The output of CASCADE_SCAN for detecting the cell wall remodeling pathway using different parameters**. Click here for file

Additional file 6 **Seed proteins and the output of CASCADE_SCAN for detecting the cell wall remodeling pathway**. Click here for file

Additional file 7 **The output of CASCADE_SCAN for detecting the osmolyte synthesis pathway using different parameters**. Click here for file

Additional file 8 **Seed proteins and the output of CASCADE_SCAN for detecting the osmolyte synthesis pathway**. Click here for file
