# Supplementary material for: CASCADE_SCAN: mining signal transduction network from high-throughput data based on steepest descent method
Source: BMC Bioinformatics. 2011 May 17;12:164. doi: 10.1186/1471-2105-12-164 (PMC3120702; doi:10.1186/1471-2105-12-164)
Supplement: Additional file 1 — The output of CASCADE_SCAN for detecting the pheromone response pathway using different parameters. [file 1471-2105-12-164-S1.PDF]

Additional file 1: The output of CASCADE_SCAN for detecting the pheromone response pathway using different parameters.

Index	Parameters for CASCADE_SCAN	Precision (%)	Recall (%)
1	PPI score threshold: 0.950, Credible PPI score threshold: 0.950, DFS path length: 5	47	71
2	PPI score threshold: 0.900, Credible PPI score threshold: 0.950, DFS path length: 5	47	73
3	PPI score threshold: 0.850, Credible PPI score threshold: 0.950, DFS path length: 5	47	73
4	PPI score threshold: 0.800, Credible PPI score threshold: 0.950, DFS path length: 5	51	74
5	PPI score threshold: 0.950, Credible PPI score threshold: 0.980, DFS path length: 5	43	71
6	PPI score threshold: 0.900, Credible PPI score threshold: 0.980, DFS path length: 5	49	70
7	PPI score threshold: 0.850, Credible PPI score threshold: 0.980, DFS path length: 5	49	71
8	PPI score threshold: 0.800, Credible PPI score threshold: 0.980, DFS path length: 5	51	73
9	PPI score threshold: 0.950, Credible PPI score threshold: 0.950, DFS path length: 2	61	75
10	PPI score threshold: 0.900, Credible PPI score threshold: 0.950, DFS path length: 2	47	81
11	PPI score threshold: 0.850, Credible PPI score threshold: 0.950, DFS path length: 2	48	78
12	PPI score threshold: 0.800, Credible PPI score threshold: 0.950, DFS path length: 2	54	75
13	PPI score threshold: 0.950, Credible PPI score threshold: 0.980, DFS path length: 2	57	76
14	PPI score threshold: 0.900, Credible PPI score threshold: 0.980, DFS path length: 2	46	80
15	PPI score threshold: 0.850, Credible PPI score threshold: 0.980, DFS path length: 2	49	74
16	PPI score threshold: 0.800, Credible PPI score threshold: 0.980, DFS path length: 2	54	75

(For each of the parameters combination, there are 20 time repeats by randomly selected four seed proteins)
