# Supplementary material for: CASCADE_SCAN: mining signal transduction network from high-throughput data based on steepest descent method
Source: BMC Bioinformatics. 2011 May 17;12:164. doi: 10.1186/1471-2105-12-164 (PMC3120702; doi:10.1186/1471-2105-12-164)
Supplement: Additional file 2 — Seed proteins and the output of CASCADE_SCAN for detecting the pheromone response pathway. [file 1471-2105-12-164-S2.PDF]

Additional file 2: seed proteins and the output of CASCADE_SCAN for detecting the pheromone response pathway.

Index	Primer number	Seed proteins (blue color) and output of CASCADE_SCAN	Precision (%)	Recall (%)
1	4	RSR1; CDC42; CDC24; BNI1; BEM1; BEM4; CLA4; GIC2; MSB1; MSB3; PEA2; SPA2; STE20; SLT2; ACT1; BOI2; CDC28; CLN2; DIG1; BOI1; RGA2; DIG2; KSS1; SSK1; FAR1; RHO1; STE12; BEM3; TEC1; STE4; STE11; STE7; SKM1; BNR1; MSG5; FUS3; GPA1; RTT109; RGA1; GIC1; RDI1; STE50; STE18; STE5; FUS2; BUD2; RHO3; FUS1;	35	85
2	4	SST2; CDC42; CDC24; BEM1; CLA4; STE20; ACT1; KSS1; FAR1; RAS2; STE12; STE4; STE11; STE7; MSG5; FUS3; GPA1; RGA1; GIC1; STE50; STE18; STE5; STE2; STE3;	67	80
3	4	RSR1; CDC42; CDC24; BNI1; BUD6; BEM1; BEM4; CLA4; GIC2; PEA2; SPA2; STE20; NIP100; ACT1; BOI2; DYN1; DIG1; RGA2; DMA2; DIG2; KSS1; FAR1; RHO1; DMA1; STE12; BEM3; TEC1; STE4; STE11; STE7; SKM1; BNR1; MSG5; FUS3; GPA1; JNM1; ARP1; RTT109; LDB18; RGA1; GIC1; KAR4; STE50; STE18; STE5; STE2; RHO3; AKR1; PFY1; FUS1;	36	90
4	4	CDC42; CDC24; BNI1; BEM1; CLA4; GIC2; LTE1; SPA2; STE20; FKH2; SLT2; NIP100; TPM1; ACT1; DYN1; SWI6; GIN4; DIG1; BUB2; DIG2; KSS1; FAR1; RAS2; GPA2; STE12; TEC1; STE4; STE11; STE7; SKM1; MSG5; FUS3; GPA1; JNM1; RGA1; RLM1; GIC1; KAR4; NDD1; MCM1; STE5; STE2; FUS2; FUS1; FLO8;	40	90
5	4	SST2; CDC42; CDC24; BEM1; CLA4; LTE1; PEA2; SPA2; STE20; ACT1; DIG1; RGA2; SSK2; BUB2; PBS2; DIG2; KSS1; SSK1; FAR1; RHO1; PTP3; CDC37; STE12; BEM3; TEC1; STE4; YPD1; STE11; STE7; SKM1; MSG5; FUS3; GPA1; RTT109; RGA1; KAR4; STE50; STE18; MCM1; STE5; MPS1; STE2; FUS2; SSU81; RHO3; AKR1; FUS1; FLO8; FLO1; FLO10;	36	90
6	4	SST2; CDC42; CDC24; BEM1; CLA4; STE20; DIG1; DIG2; KSS1; FAR1; STE12; STE4; STE11; STE7; FUS3; GPA1; STE50; STE18; STE5;	79	75
7	4	CDC42; CDC24; BEM1; CLA4; SPA2; STE20; ACT1; RGA2; KSS1; FAR1; RHO1; BEM3; STE4; STE11; STE7; MSG5; FUS3; GPA1; RGA1; GIC1; STE50; STE18; STE5; RHO3;	54	65
8	4	SST2; STE20; DIG1; DIG2; KSS1; STE12; STE4; STE11; STE7; MSG5; FUS3; GPA1; KAR4; STE50; STE18; STE5; STE2; STE3;	78	70
9	3	RSR1; CDC42; CDC24; BNI1; SWE1; BEM1; CLA4; GIC2; LTE1; SPA2; STE20; FKH2; NIP100; TPM1; ACT1; CDC28; CLN2; DYN1; KEL1; DIG1; BUB2; FKH1; DIG2; KSS1; FAR1; RAS2; RAS1; GPA2; CLB2; STE12; CDC15; TEC1; STE4; STE11; STE7; SKM1; FUS3; JNM1; RGA1; GIC1; KAR4; MCM1; STE5; STE2; FUS2; ARG82; FUS1; FLO8;	29	70

10	4	CDC42; CDC24; BNI1; BUD6; BEM1; CLA4; PEA2; SPA2; STE20; TPM1; ACT1; MYO2; RGA2; RVS161; KSS1; FAR1; RHO1; BEM3; STE4; STE11; STE7; BNR1; FUS3; GPA1; RGA1; GIC1; STE50; STE18; STE5; FUS2; RHO3; FUS1;	41	65
11	4	RSR1; CDC42; CDC24; BNI1; BEM1; CLA4; GIC2; LTE1; MSB3; PEA2; SPA2; STE20; BEM2; NIP100; TPM1; ACT1; CDC3; CDC10; CDC11; CDC12; DYN1; MYO2; RGA2; BUB2; RVS161; FAR1; RHO1; ELM1; BEM3; STE4; STE11; STE7; SKM1; BNR1; FUS3; GPA1; ARP1; SAC7; RGA1; GIC1; RDI1; MSB2; STE18; STE5; TOR2; BUD4; GCS1; RHO3; TPM2; KIN4;	26	65
12	4	SST2; RSR1; CDC42; CDC24; BEM1; BEM4; CLA4; GIC2; PEA2; SPA2; STE20; SLT2; ACT1; BOI2; DIG1; RGA2; SSK2; PBS2; DIG2; KSS1; SSK1; FAR1; RHO1; CDC37; STE12; BEM3; TEC1; STE4; YPD1; STE11; STE7; SLN1; SKM1; MSG5; FUS3; GPA1; RTT109; RGA1; GIC1; STE50; STE18; STE5; MPT5; MPS1; STE2; SSU81; BUD2; RHO3; AKR1; FUS1;	34	85
13	4	SST2; STE20; DIG1; DIG2; KSS1; RAS2; STE12; TEC1; STE4; STE11; STE7; FUS3; GPA1; STE50; STE18; STE5; STE2;	71	60
14	4	CDC42; CDC24; BEM1; CLA4; STE20; DIG1; DIG2; KSS1; FAR1; STE12; STE4; STE11; STE7; FUS3; GPA1; STE50; STE18; STE5;	83	75
15	4	RSR1; CDC42; CDC24; BEM1; BEM4; CLA4; GIC2; LTE1; MSB1; MSB3; PEA2; SPA2; STE20; ACT1; BOI2; DIG1; BOI1; RGA2; SSK2; BUB2; PBS2; DIG2; KSS1; SSK1; FAR1; RHO1; CDC37; STE12; BEM3; TEC1; STE4; YPD1; STE11; STE7; SKM1; MSG5; FUS3; GPA1; RTT109; RGA1; GIC1; KAR4; STE50; STE18; STE5; MPS1; SSU81; RHO3; FUS1;	33	80
16	4	SST2; STE20; DIG1; DIG2; KSS1; FAR1; STE12; STE4; STE11; STE7; MSG5; FUS3; GPA1; STE50; STE18; STE5; STE2;	82	70
17	4	SST2; CDC42; CDC24; CLA4; STE20; DIG1; DIG2; KSS1; FAR1; RAS2; STE12; STE4; STE11; STE7; MSG5; FUS3; GPA1; STE50; STE18; STE5;	75	75
18	4	SST2; CDC42; CDC24; CLA4; STE20; DIG1; DIG2; KSS1; SSK1; FAR1; STE12; BEM3; TEC1; STE4; STE11; STE7; SKM1; FUS3; GPA1; RGA1; KAR4; STE50; STE18; STE5; STE2;	60	75
19	4	SST2; CLA4; STE20; DIG1; DIG2; KSS1; STE12; STE4; STE11; STE7; MSG5; FUS3; GPA1; STE50; STE18; STE5; STE2; STE3;	78	70
20	4	RSR1; CDC42; CDC24; BNI1; SWE1; BEM1; CLA4; GIC2; LTE1; SPA2; STE20; FKH2; NIP100; TPM1; ACT1; CDC28; CLN2; DYN1; KEL1; DIG1; BUB2; FKH1; DIG2; KSS1; FAR1; RAS2; RAS1; GPA2; CLB2; STE12; CDC15; TEC1; STE4; STE11; STE7; SKM1; FUS3; JNM1; RGA1; GIC1; KAR4; MCM1; STE5; STE2; FUS2; ARG82; FUS1; FLO8;	33	80
average			54	76

(PPI score threshold: 0.800, credible PPI score threshold: 0.980, DFS path length: 2)
