# Supplementary material for: CASCADE_SCAN: mining signal transduction network from high-throughput data based on steepest descent method
Source: BMC Bioinformatics. 2011 May 17;12:164. doi: 10.1186/1471-2105-12-164 (PMC3120702; doi:10.1186/1471-2105-12-164)
Supplement: Additional file 3 — The output of CASCADE_SCAN for detecting the filamentous growth pathway using different parameters. [file 1471-2105-12-164-S3.PDF]

Additional file 3: The output of CASCADE_SCAN for detecting the filamentous growth pathway using different parameters.

Index	Parameters forCASCADE_SCAN	Precision (%)	Recall (%)
1	PPI score threshold: 0.950, Credible PPI score threshold: 0.950, DFS path length: 5	22	93
2	PPI score threshold: 0.900, Credible PPI score threshold: 0.950, DFS path length: 5	36	89
3	PPI score threshold: 0.850, Credible PPI score threshold: 0.950, DFS path length: 5	23	95
4	PPI score threshold: 0.800, Credible PPI score threshold: 0.950, DFS path length: 5	22	98
5	PPI score threshold: 0.950, Credible PPI score threshold: 0.980, DFS path length: 5	21	93
6	PPI score threshold: 0.900, Credible PPI score threshold: 0.980, DFS path length: 5	32	91
7	PPI score threshold: 0.850, Credible PPI score threshold: 0.980, DFS path length: 5	22	95
8	PPI score threshold: 0.800, Credible PPI score threshold: 0.980, DFS path length: 5	21	93
9	PPI score threshold: 0.950, Credible PPI score threshold: 0.950, DFS path length: 2	26	98
10	PPI score threshold: 0.900, Credible PPI score threshold: 0.950, DFS path length: 2	24	98
11	PPI score threshold: 0.850, Credible PPI score threshold: 0.950, DFS path length: 2	23	98
12	PPI score threshold: 0.800, Credible PPI score threshold: 0.950, DFS path length: 2	22	98
13	PPI score threshold: 0.950, Credible PPI score threshold: 0.980, DFS path length: 2	26	96
14	PPI score threshold: 0.900, Credible PPI score threshold: 0.980, DFS path length: 2	22	95
15	PPI score threshold: 0.850, Credible PPI score threshold: 0.980, DFS path length: 2	22	95
16	PPI score threshold: 0.800, Credible PPI score threshold: 0.980, DFS path length: 2	21	93

(For each of the parameters combination, there are 20 time repeats by randomly selected three or four seed proteins from SSU81, RAS2, KSS1 and TEC1)
