# Supplementary material for: CASCADE_SCAN: mining signal transduction network from high-throughput data based on steepest descent method
Source: BMC Bioinformatics. 2011 May 17;12:164. doi: 10.1186/1471-2105-12-164 (PMC3120702; doi:10.1186/1471-2105-12-164)
Supplement: Additional file 4 — Seed proteins and the output of CASCADE_SCAN for detecting the filamentous growth pathway. [file 1471-2105-12-164-S4.PDF]

Additional file 4: seed proteins and the output of CASCADE_SCAN for detecting the filamentous growth pathway.

Index	Primer number	Seed proteins (blue color) and output of CASCADE_SCAN	Precision (%)	Recall (%)
1	3	CDC42; CDC24; BNI1; BUD6; CLA4; LTE1; PEA2; SPA2; STE20; CHS5; ACT1; DYN1; DIG1; RGA2; SSK2; BUB2; PBS2; DIG2; KSS1; SSK1; FAR1; RHO1; CDC37; STE12; BEM3; HOG1; TEC1; STE4; YPD1; STE11; STE7; SLN1; SKM1; BNR1; MSG5; FUS3; GPA1; RTT109; RGA1; RDI1; KAR4; STE50; STE18; STE5; MPS1; STE2; FUS2; SSU81; RHO3; FUS1;	20	91
2	3	PEA2; SPA2; STE20; DIG1; DIG2; KSS1; RAS2; STE12; STE4; STE11; STE7; FUS3; KAR4; STE50; STE5; FUS2; SSU81; FUS1;	50	82
3	3	CDC42; CDC24; CLA4; STE20; CYR1; DIG1; CDC25; TPK2; BCY1; TPK3; IRA2; TPK1; SSK2; PBS2; DIG2; KSS1; SSK1; RAS2; RAS1; MSI1; GPA2; STE12; BEM3; HOG1; TEC1; STE4; YPD1; IRA1; STE11; STE7; SLN1; SKM1; FUS3; PDE2; RGA1; RDI1; KAR4; MSB2; STE50; MCM1; STE5; STE2; SSU81; FUS1; FLO8; FLO1; FLO10;	23	100
4	3	CLA4; STE20; CYR1; DIG1; CDC25; TPK2; BCY1; TPK3; IRA2; TPK1; DIG2; KSS1; SSK1; FAR1; RAS2; RAS1; MSI1; GPA2; STE12; TEC1; STE4; IRA1; STE11; STE7; SKM1; MSG5; FUS3; GPA1; RTT109; KAR4; STE50; STE18; STE5; STE2; FUS1;	26	82
5	4	SPA2; STE20; DIG1; DIG2; KSS1; RAS2; STE12; TEC1; STE4; STE11; STE7; FUS3; STE50; STE5; FUS2; SSU81; FUS1;	59	91
average			36	89

(PPI score threshold: 0.900, credible PPI score threshold: 0.950, DFS path length: 5)
