# Supplementary material for: CASCADE_SCAN: mining signal transduction network from high-throughput data based on steepest descent method
Source: BMC Bioinformatics. 2011 May 17;12:164. doi: 10.1186/1471-2105-12-164 (PMC3120702; doi:10.1186/1471-2105-12-164)
Supplement: Additional file 5 — The output of CASCADE_SCAN for detecting the cell wall remodeling pathway using different parameters. [file 1471-2105-12-164-S5.PDF]

Additional file 5: The output of CASCADE_SCAN for detecting the cell wall remodeling pathway using different parameters.

Index	Parameters for CASCADE_SCAN	Precision (%)	Recall (%)
1	PPI score threshold: 0.950, Credible PPI score threshold: 0.950, DFS path length: 5	22	72
2	PPI score threshold: 0.900, Credible PPI score threshold: 0.950, DFS path length: 5	21	71
3	PPI score threshold: 0.850, Credible PPI score threshold: 0.950, DFS path length: 5	20	67
4	PPI score threshold: 0.800, Credible PPI score threshold: 0.950, DFS path length: 5	21	71
5	PPI score threshold: 0.950, Credible PPI score threshold: 0.980, DFS path length: 5	22	71
6	PPI score threshold: 0.900, Credible PPI score threshold: 0.980, DFS path length: 5	21	70
7	PPI score threshold: 0.850, Credible PPI score threshold: 0.980, DFS path length: 5	20	67
8	PPI score threshold: 0.800, Credible PPI score threshold: 0.980, DFS path length: 5	21	69
9	PPI score threshold: 0.950, Credible PPI score threshold: 0.950, DFS path length: 2	29	47
10	PPI score threshold: 0.900, Credible PPI score threshold: 0.950, DFS path length: 2	19	55
11	PPI score threshold: 0.850, Credible PPI score threshold: 0.950, DFS path length: 2	21	69
12	PPI score threshold: 0.800, Credible PPI score threshold: 0.950, DFS path length: 2	21	67
13	PPI score threshold: 0.950, Credible PPI score threshold: 0.980, DFS path length: 2	29	50
14	PPI score threshold: 0.900, Credible PPI score threshold: 0.980, DFS path length: 2	19	55
15	PPI score threshold: 0.850, Credible PPI score threshold: 0.980, DFS path length: 2	21	68
16	PPI score threshold: 0.800, Credible PPI score threshold: 0.980, DFS path length: 2	20	65

(For each of the parameters combination, there are 20 time repeats by randomly selected three seed proteins)
