# Supplementary material for: CASCADE_SCAN: mining signal transduction network from high-throughput data based on steepest descent method
Source: BMC Bioinformatics. 2011 May 17;12:164. doi: 10.1186/1471-2105-12-164 (PMC3120702; doi:10.1186/1471-2105-12-164)
Supplement: Additional file 6 — Seed proteins and the output of CASCADE_SCAN for detecting the cell wall remodeling pathway. [file 1471-2105-12-164-S6.PDF]

Additional file 6: seed proteins and the output of CASCADE_SCAN for detecting the cell wall remodeling pathway.

Index	Primer number	Seed proteins (blue color) and output of CASCADE_SCAN	Precision (%)	Recall (%)
1	3	CDC42; CDC24; CLA4; LTE1; SWI4; STE20; BCK1; SLT2; ROM2; SLG1; RCK2; PKC1; SSK2; BUB2; PBS2; KSS1; SSK1; RHO1; PTP3; CDC37; STE12; BEM3; HOG1; STE4; YPD1; STE11; STE7; SLN1; PTP2; SKM1; MSG5; FUS3; GPA1; NBP2; FPS1; RGA1; MID2; RDI1; KAR4; MSB2; STE50; STE18; SKO1; STE5; MPS1; SSU81; RHO3; MKK1; FUS1; WSC2;	18	60
2	3	CDC42; CDC24; CLA4; PEA2; SPA2; SWI4; STE20; BCK1; SLT2; SLG1; RCK2; SWI6; PKC1; SSK2; PBS2; KSS1; SSK1; RHO1; MKK2; PTP3; CDC37; STE12; BEM3; HOG1; STE4; YPD1; STE11; STE7; SDP1; SLN1; PTP2; SKM1; MSG5; FUS3; GPA1; FPS1; RGA1; MID2; RLM1; RDI1; MSB2; STE50; STE18; SKO1; STE5; MPS1; SSU81; RHO3; MKK1; FUS1;	22	73
3	3	CDC40; PRP45; PRP8; CDC42; CDC24; BUD6; CLA4; PEA2; SPA2; SWI4; STE20; BCK1; CHS5; SLT2; SMI1; FKS1; ROM2; SLG1; ACT1; AAR2; NAB2; ZDS1; ACK1; SWI6; PKC1; SLU7; PRP18; MLP1; RGA2; CMP2; CNB1; CNA1; RHO1; MKK2; MLP2; BEM3; SDP1; BNR1; MSG5; RGA1; GSC2; RLM1; GIC1; RDI1; GFD1; PRP16; FUS2; RHO3; MKK1; FUS1;	22	73
4	3	PAF1; CDC42; CDC24; BUD6; PEA2; SPA2; SWI4; BCK1; CHS5; SLT2; RGD1; ROM2; SLG1; CDC48; SIN4; ACT1; CDC28; CKS1; CLN2; MBP1; SWI6; ORC6; PKC1; FAR3; RGA2; CNB1; RHO1; MKK2; CLB2; PTP3; BEM3; BCK2; SDP1; MSG5; CLB5; RGA1; MID2; RDI1; CLN3; WSC3; NDD1; STB1; TOR1; FUS2; RHO3; MKK1; FUS1; WHI5; WSC2; CLB6;	22	73
5	3	CDC42; CDC24; BNI1; BUD6; BEM1; BEM4; CLA4; GIC2; PEA2; SPA2; SWI4; STE20; BCK1; CHS5; SLT2; NIP100; SMI1; FKS1; RGD1; ROM2; SLG1; ACT1; CDC28; DYN1; MBP1; SWI6; PKC1; RGA2; RHO1; MKK2; BEM3; SDP1; BNR1; MSG5; ARP1; SAC7; RGA1; GSC2; MID2; RLM1; GIC1; RDI1; FUS2; RHO3; MKK1; FUS1; GYP7;	23	73
6	3	CDC40; SNU114; CDC42; CLA4; PEA2; SPA2; STE20; BCK1; SLT2; RCK2; DHH1; SWI6; PKC1; PRP18; MLP1; SSK2; PBS2; KSS1; SSK1; MKK2; MLP2; PTP3; CDC37; STE12; BEM3; HOG1; STE4; YPD1; STE11; STE7; SDP1; SLN1; PTP2; SKM1; MSG5; FUS3; GPA1; FPS1; RGA1; RLM1; RDI1; MSB2; STE50; STE18; SKO1; STE5; MPS1; SSU81; MKK1; FUS1;	18	60
7	3	CDC42; CDC24; BNI1; BUD6; BEM1; BEM4; CLA4; GIC2; PEA2; SPA2; SWI4; STE20; BCK1; BEM2; CHS5; SLT2; SMI1; FKS1; RGD1; ROM2; SLG1; ACT1; CDC28; IPP1; ACK1; MBP1; SWI6; PKC1; RGA2; FAR1; RHO1; MKK2; BEM3; SDP1; BNR1;	26	87

		PPZ1; MSG5; RGA1; GSC2; MID2; RLM1; GIC1; RDI1; CLN3; WSC3; FUS2; RHO3; MKK1; FUS1; WSC2;		
8	3	CDC42; CDC24; SWE1; BUD6; PEA2; SPA2; SWI4; BCK1; CHS5; SLT2; SMI1; RGD1; ROM2; SLG1; ACT1; CDC28; CLN2; CLB3; SIC1; MBP1; SWI6; ORC5; PKC1; CDC6; FAR3; RGA2; CDC5; RHO1; MKK2; CLB2; BEM3; SDP1; MSG5; CDH1; CLB5; CLB4; RGA1; MID2; RLM1; RDI1; CLB1; CLN3; WSC3; FUS2; RHO3; MKK1; FUS1; WHI5; WSC2; CLB6;	24	80
9	3	CDC42; CDC24; CLA4; LTE1; STE20; BCK1; SLT2; SLG1; RCK2; PKC1; PTC1; RGA2; SSK2; BUB2; PBS2; KSS1; SSK1; MKK2; PTP3; CDC37; STE12; BEM3; HOG1; STE4; YPD1; STE11; STE7; SDP1; SLN1; PTP2; SKM1; MSG5; FUS3; GPA1; NBP2; FPS1; RGA1; RDI1; KAR4; MSB2; STE50; STE18; SKO1; STE5; MPS1; SSU81; RHO3; MKK1; FUS1; WSC2;	16	53
10	3	CDC40; CLF1; PRP19; PRP45; PRP8; SYF1; SWI4; BCK1; SLT2; SMI1; RGD1; ROM2; SLG1; AAR2; NAB2; ENO2; ENO1; CDC28; CKS1; CLN2; ZDS1; ACK1; PGK1; MBP1; SWI6; PKC1; SLU7; PRP18; MLP1; FAR3; RHO1; MKK2; MLP2; CLB2; SDP1; MSG5; MID2; GFD1; CLN3; WSC3; TOR2; PRP16; RHO3; MKK1; LRG1; WHI5; WSC2; CLB6;	25	80
11	3	CDC40; CDC42; CDC24; BNI1; BUD6; BEM1; BEM4; CLA4; GIC2; PEA2; SPA2; SWI4; STE20; BCK1; CHS5; SLT2; SMI1; SLG1; ACT1; NAB2; CDC28; ZDS1; DYN1; MBP1; SWI6; PKC1; SLU7; PRP18; MLP1; RGA2; FAR1; RHO1; MKK2; MLP2; BEM3; STE4; SDP1; BNR1; PPZ1; MSG5; RGA1; RLM1; GIC1; RDI1; GFD1; CLN3; FUS2; RHO3; MKK1; FUS1;	20	67
12	3	PAF1; CDC40; CDC42; CDC24; BNI1; BUD6; BEM1; BEM4; CLA4; GIC2; MSB3; PEA2; SPA2; SWI4; STE20; BCK1; CHS5; SLT2; SMI1; STO1; HPR1; ACT1; CDC28; DYN1; MBP1; SWI6; PKC1; SLU7; PRP18; MLP1; RGA2; FAR1; RHO1; MKK2; MLP2; BEM3; STE4; SDP1; BNR1; PPZ1; MSG5; RGA1; RLM1; GIC1; RDI1; CLN3; FUS2; RHO3; MKK1; FUS1;	18	60
13	3	CDC42; CDC24; BNI1; BUD6; BEM1; BEM4; CLA4; GIC2; MSB1; MSB3; PEA2; SPA2; SWI4; STE20; BCK1; CHS5; SLT2; SMI1; RGD1; ROM2; SLG1; ACT1; ENO2; ENO1; CDC28; DYN1; MBP1; SWI6; PKC1; RGA2; FAR1; RHO1; MKK2; CLB2; BEM3; STE4; SDP1; BNR1; PPZ1; MSG5; RGA1; MID2; RLM1; GIC1; RDI1; CLN3; FUS2; RHO3; MKK1; FUS1;	20	67
14	3	CDC42; CDC24; BNI1; BUD6; BEM1; BEM4; CLA4; GIC2; MSB1; MSB3; PEA2; SPA2; SWI4; STE20; BCK1; CHS5; SLT2; SMI1; SLG1; ACT1; CDC28; CDC10; CDC12; DYN1; MBP1; SWI6; PKC1; RGA2; FAR1; RHO1; MKK2; CLB2; BEM3; STE4; SDP1; BNR1; PPZ1; MSG5; ARP1; RGA1; RLM1; GIC1; RDI1; CLN3; FUS2; RHO3; MKK1; FUS1; WSC2; GYP7;	20	67

15	3	CDC42; CDC24; BNI1; BUD6; BEM1; BEM4; CLA4; GIC2; MSB1; MSB3; PEA2; SPA2; SWI4; STE20; BCK1; CHS5; SLT2; SMI1; RGD1; ROM2; SLG1; ACT1; ENO2; ENO1; CDC28; DYN1; MBP1; SWI6; PKC1; RGA2; FAR1; RHO1; MKK2; CLB2; BEM3; STE4; SDP1; BNR1; PPZ1; MSG5; RGA1; MID2; RLM1; GIC1; RDI1; CLN3; FUS2; RHO3; MKK1; FUS1;	20	67
16	3	CDC40; PRP19; PRP45; PRP8; SYF1; SWI4; BCK1; SLT2; SMI1; RGD1; ROM2; SLG1; SIN4; AAR2; NAB2; ENO2; ENO1; CDC28; CKS1; CLN2; ZDS1; PGK1; MBP1; SWI6; ORC6; PKC1; SLU7; PRP18; MLP1; FAR3; RHO1; MKK2; MLP2; CLB2; SDP1; MSG5; CLB5; MID2; GFD1; CLN3; WSC3; NDD1; STB1; TOR2; PRP16; RHO3; MKK1; WHI5; WSC2; CLB6;	24	80
17	3	CDC42; CDC24; BNI1; BUD6; BEM1; BEM4; CLA4; GIC2; MSB1; MSB3; PEA2; SPA2; SWI4; STE20; BCK1; CHS5; SLT2; SMI1; RGD1; ROM2; SLG1; ACT1; ENO2; ENO1; CDC28; DYN1; MBP1; SWI6; PKC1; RGA2; FAR1; RHO1; MKK2; CLB2; BEM3; STE4; SDP1; BNR1; PPZ1; MSG5; RGA1; MID2; RLM1; GIC1; RDI1; CLN3; FUS2; RHO3; MKK1; FUS1;	20	67
18	3	CDC42; CDC24; BUD6; CLA4; PEA2; SPA2; SWI4; STE20; BCK1; CHS5; SLT2; SMI1; SLG1; RCK2; ACT1; SWI6; PKC1; RGA2; SSK2; PBS2; KSS1; SSK1; MKK2; PTP3; CDC37; BEM3; HOG1; STE4; YPD1; STE11; STE7; SDP1; SLN1; PTP2; SKM1; MSG5; FUS3; RGA1; RLM1; RDI1; MSB2; STE50; STE5; MPS1; FUS2; SSU81; RHO3; MKK1; FUS1; WSC2;	22	73
19	3	CDC42; CDC24; CLA4; SPA2; STE20; BCK1; SLT2; FKS1; ROM2; SLG1; RCK2; SWI6; PKC1; RGA2; SSK2; PBS2; KSS1; SSK1; RHO1; MKK2; PTP3; CDC37; STE12; BEM3; HOG1; STE4; YPD1; STE11; STE7; SLN1; PTP2; SKM1; MSG5; FUS3; GPA1; SAC7; RGA1; GSC2; MID2; RLM1; RDI1; MSB2; STE50; STE18; STE5; MPS1; SSU81; RHO3; MKK1; FUS1;	22	73
20	3	CDC42; CLA4; SWI4; STE20; BCK1; SLT2; RCK2; CDC28; CLN2; MBP1; SWI6; PKC1; FAR3; SSK2; PBS2; KSS1; SSK1; MKK2; CLB2; PTP3; CDC37; STE12; BEM3; HOG1; STE4; YPD1; STE11; STE7; SDP1; SLN1; PTP2; SKM1; MSG5; FUS3; GPA1; FPS1; RGA1; RDI1; CLN3; MSB2; STE50; STE18; SKO1; STE5; MPS1; SSU81; MKK1; FUS1; WHI5; CLB6;	16	53
average			21	69

(PPI score threshold: 0.800, credible PPI score threshold: 0.980, DFS path length: 5)
