# Supplementary material for: CASCADE_SCAN: mining signal transduction network from high-throughput data based on steepest descent method
Source: BMC Bioinformatics. 2011 May 17;12:164. doi: 10.1186/1471-2105-12-164 (PMC3120702; doi:10.1186/1471-2105-12-164)
Supplement: Additional file 7 — The output of CASCADE_SCAN for detecting the osmolyte synthesis pathway using different parameters. [file 1471-2105-12-164-S7.PDF]

Additional file 7: The output of CASCADE_SCAN for detecting the osmolyte synthesis pathway using different parameters.

Index	Parameters for CASCADE_SCAN	Precision (%)	Recall (%)
1	PPI score threshold: 0.950, Credible PPI score threshold: 0.950, DFS path length: 5	14	57
2	PPI score threshold: 0.900, Credible PPI score threshold: 0.950, DFS path length: 5	21	77
3	PPI score threshold: 0.850, Credible PPI score threshold: 0.950, DFS path length: 5	21	77
4	PPI score threshold: 0.800, Credible PPI score threshold: 0.950, DFS path length: 5	20	77
5	PPI score threshold: 0.950, Credible PPI score threshold: 0.980, DFS path length: 5	14	56
6	PPI score threshold: 0.900, Credible PPI score threshold: 0.980, DFS path length: 5	20	77
7	PPI score threshold: 0.850, Credible PPI score threshold: 0.980, DFS path length: 5	20	77
8	PPI score threshold: 0.800, Credible PPI score threshold: 0.980, DFS path length: 5	20	77
9	PPI score threshold: 0.950, Credible PPI score threshold: 0.950, DFS path length: 2	25	60
10	PPI score threshold: 0.900, Credible PPI score threshold: 0.950, DFS path length: 2	18	65
11	PPI score threshold: 0.850, Credible PPI score threshold: 0.950, DFS path length: 2	20	79
12	PPI score threshold: 0.800, Credible PPI score threshold: 0.950, DFS path length: 2	19	78
13	PPI score threshold: 0.950, Credible PPI score threshold: 0.980, DFS path length: 2	26	60
14	PPI score threshold: 0.900, Credible PPI score threshold: 0.980, DFS path length: 2	18	65
15	PPI score threshold: 0.850, Credible PPI score threshold: 0.980, DFS path length: 2	20	79
16	PPI score threshold: 0.800, Credible PPI score threshold: 0.980, DFS path length: 2	19	78

(For each of the parameters combination, there are 20 time repeats by randomly selected three seed proteins)
