# Supplementary material for: CASCADE_SCAN: mining signal transduction network from high-throughput data based on steepest descent method
Source: BMC Bioinformatics. 2011 May 17;12:164. doi: 10.1186/1471-2105-12-164 (PMC3120702; doi:10.1186/1471-2105-12-164)
Supplement: Additional file 8 — Seed proteins and the output of CASCADE_SCAN for detecting the osmolyte synthesis pathway. [file 1471-2105-12-164-S8.PDF]

Additional file 8: seed proteins and the output of CASCADE_SCAN for detecting the osmolyte synthesis pathway.

Index	Primer number	Seed proteins (blue color) and output of CASCADE_SCAN	Precision (%)	Recall (%)
1	3	CDC42; CLA4; STE20; PRE9; RCK2; RPN1; PRE2; PRE1; PRE10; PRE3; PRE5; PRE6; PRE7; PRE8; PUP1; PUP2; SCL1; PRE4; PUP3; PTC1; SSK2; PBS2; SSK1; HOG1; YPD1; STE11; SLN1; SKM1; SSK22; NBP2; STB5; SRL2; SMP1; SKN7; SSU81; PTK1;	25	75
2	3	PRE9; BMH1; RCK2; RPN1; PRE2; PRE1; PRE10; PRE3; PRE5; PRE6; PRE7; PRE8; PUP1; PUP2; SCL1; BMH2; HOT1; PRE4; PUP3; SNF1; PTC1; HSP104; IRA2; RIM15; SSK2; PBS2; SSK1; RAS2; RAS1; GPA2; SGD1; PTP3; HOG1; MSN4; YPD1; IRA1; STE11; PTP2; SSK22; FUS3; NBP2; FPS1; STE50; MSN2; SKO1; SMP1; HSF1; SSU81; ACM1; SCH9;	18	75
3	3	CDC42; CDC24; SWE1; BEM1; CLA4; LTE1; PEA2; SPA2; STE20; ACT1; DIG1; RGA2; SSK2; BUB2; PBS2; DIG2; KSS1; SSK1; RHO1; CDC37; STE12; BEM3; STE4; YPD1; STE11; STE7; SLN1; SKM1; SSK22; FUS3; GPA1; RGA1; GIC1; RDI1; KAR4; STE50; STE18; STE5; MPS1; SKN7; STE2; FUS2; SSU81; RHO3; AKR1; FUS1; KIN4;	17	67
4	3	CDC42; CDC24; SWE1; BEM1; CLA4; LTE1; PEA2; SPA2; STE20; FKH2; ACT1; DIG1; RGA2; SSK2; BUB2; PBS2; FKH1; DIG2; KSS1; SSK1; RHO1; CDC37; STE12; BEM3; STE4; YPD1; STE11; STE7; SLN1; SKM1; FUS3; GPA1; RGA1; RDI1; ARGR2; KAR4; STE50; STE18; NDD1; MCM1; STE5; MPS1; STE2; FUS2; SSU81; RHO3; ARG82; AKR1; FUS1; MATA2;	16	67
5	3	CDC42; CDC24; CLA4; LTE1; STE20; RCK2; ACT1; DIG1; PTC1; RGA2; RIM15; SSK2; BUB2; PBS2; DIG2; KSS1; SSK1; PTP3; CDC37; STE12; BEM3; HOG1; STE4; MSN4; YPD1; STE11; STE7; SLN1; PTP2; SKM1; FUS3; GPA1; NBP2; FPS1; RGA1; RDI1; KAR4; MSB2; STE50; STE18; MSN2; SKO1; STE5; MPS1; STE2; FUS2; SSU81; RHO3; AKR1; FUS1;	20	83
6	4	CDC42; CDC24; CLA4; LTE1; STE20; RCK2; PTC1; RGA2; SSK2; BUB2; PBS2; KSS1; SSK1; PTP3; CDC37; STE12; BEM3; HOG1; STE4; YPD1; STE11; STE7; SLN1; PTP2; SKM1; FUS3; GPA1; NBP2; FPS1; RGA1; RDI1; KAR4; SRL2; MSB2; STE50; STE18; SKO1; STE5; SMP1; MPS1; SKN7; STE2; FUS2; SSU81; RHO3; AKR1; FUS1; PTK1;	17	67
7	4	CDC42; CDC24; CLA4; LTE1; STE20; RCK2; HSP104; RIM15; SSK2; PBS2; KSS1; SSK1; PTP3; CDC37; STE12; BEM3; HOG1; STE4; MSN4; YPD1; STE11; STE7; SLN1; PTP2; SKM1; SSK22; FUS3; GPA1; FPS1; RGA1; RDI1; MSB2; STE50; STE18; MSN2; SKO1; STE5; MPS1; HSF1; SSU81; RHO3; FUS1; SCH9;	26	92
8	4	CDC42; CDC24; CLA4; STE20; FKH2; RCK2; DIG1; SSK2;	20	75

		PBS2; FKH1; DIG2; KSS1; SSK1; PTP3; CDC37; STE12; BEM3; HOG1; STE4; YPD1; STE11; STE7; SLN1; PTP2; SKM1; FUS3; GPA1; FPS1; RGA1; RDI1; ARGR2; KAR4; MSB2; STE50; STE18; NDD1; SKO1; MCM1; STE5; MPS1; SKN7; SSU81; RHO3; ARG82; FUS1; MATA2;		
9	4	CDC42; CDC24; SWE1; BEM1; CLA4; LTE1; PEA2; SPA2; STE20; ACT1; DIG1; RGA2; RIM15; SSK2; BUB2; PBS2; DIG2; KSS1; SSK1; RHO1; CDC37; STE12; BEM3; HOG1; STE4; MSN4; YPD1; STE11; STE7; SLN1; SKM1; SSK22; FUS3; GPA1; RGA1; RDI1; KAR4; STE50; STE18; MSN2; STE5; MPS1; STE2; FUS2; SSU81; RHO3; AKR1; FUS1; SCH9; KIN4;	22	92
10	4	CDC42; CDC24; BEM1; CLA4; LTE1; SPA2; STE20; RCK2; ACT1; DIG1; PTC1; RGA2; SSK2; BUB2; PBS2; DIG2; KSS1; SSK1; RHO1; PTP3; CDC37; STE12; BEM3; HOG1; STE4; YPD1; STE11; STE7; SLN1; PTP2; SKM1; SSK22; FUS3; GPA1; FPS1; RGA1; RDI1; KAR4; STE50; STE18; SKO1; STE5; SMP1; MPS1; STE2; FUS2; SSU81; RHO3; AKR1; FUS1;	18	75
11	4	CDC42; CDC24; CLA4; LTE1; STE20; SKT5; BMH1; RCK2; SUP35; BMH2; SNF1; SSA1; TPK2; BCY1; TPK3; HSP104; IRA2; TPK1; RIM15; SSK2; STI1; BUB2; PBS2; SSK1; RAS2; RAS1; MSI1; GPA2; BEM3; MSN4; YPD1; IRA1; STE11; SLN1; PTP2; SKM1; CPR7; RGA1; STB5; RDI1; SRL2; MSN2; SIP4; SKN7; HSF1; SSU81; RHO3; ACM1; SCH9; PTK1;	18	75
12	4	CDC42; CDC24; CLA4; LTE1; STE20; SKT5; BMH1; RCK2; BMH2; REG1; HOT1; PTC1; IRA2; RIM15; SSK2; BUB2; PBS2; SSK1; RAS2; RAS1; GPA2; SGD1; PTP3; BEM3; HOG1; MSN4; YPD1; IRA1; STE11; SLN1; PTP2; SKM1; FUS3; NBP2; FPS1; RGA1; STB5; RDI1; SRL2; STE50; MSN2; SKO1; SMP1; SKN7; HSF1; SSU81; RHO3; ACM1; SCH9; PTK1;	20	83
13	4	CDC42; CDC24; BEM1; CLA4; LTE1; SPA2; STE20; FKH2; ACT1; DIG1; RGA2; SSK2; BUB2; PBS2; FKH1; DIG2; KSS1; SSK1; RHO1; CDC37; STE12; BEM3; HOG1; STE4; YPD1; STE11; STE7; SLN1; SKM1; FUS3; GPA1; NBP2; RGA1; RDI1; ARGR2; KAR4; MSB2; STE50; STE18; NDD1; MCM1; STE5; MPS1; STE2; FUS2; SSU81; RHO3; ARG82; AKR1; FUS1;	18	75
14	4	CDC42; CDC24; SWE1; BEM1; CLA4; LTE1; PEA2; SPA2; STE20; ACT1; DIG1; RGA2; SSK2; BUB2; PBS2; DIG2; KSS1; SSK1; RHO1; CDC37; STE12; BEM3; HOG1; STE4; YPD1; STE11; STE7; SLN1; SKM1; FUS3; GPA1; NBP2; RGA1; GIC1; RDI1; KAR4; MSB2; STE50; STE18; STE5; MPS1; SKN7; STE2; FUS2; SSU81; RHO3; AKR1; FUS1; KIN4;	16	67
15	4	CDC42; CLA4; STE20; PRE9; BMH1; RCK2; RPN1; PRE2; PRE1; PRE10; PRE3; PRE5; PRE6; PRE7; PRE8; PUP1; PUP2; SCL1; BMH2; PRE4; PUP3; RIM15; SSK2; PBS2; SSK1; RAS2; RAS1;	24	83

		GPA2; MSN4; YPD1; STE11; SLN1; SKM1; SSK22; RDI1; SRL2; MSN2; HSF1; SSU81; ACM1; SCH9; PTK1;		
16	4	CDC42; CDC24; CLA4; STE20; FKH2; BMH1; RCK2; BMH2; DIG1; RIM15; SSK2; PBS2; FKH1; DIG2; KSS1; SSK1; RAS2; CLB2; STE12; BEM3; TEC1; MSN4; YPD1; STE11; STE7; SLN1; SKM1; FUS3; RGA1; STB5; RDI1; ARGR2; KAR4; SRL2; NDD1; MSN2; MCM1; SKN7; STE2; HSF1; SSU81; ARG82; ARGR1; ACM1; FLO8; SCH9; PTK1; FLO1; FLO10; MATA2;	20	83
17	4	CDC42; CDC24; CLA4; STE20; FKH2; RCK2; HOT1; DIG1; PTC1; SSK2; PBS2; FKH1; DIG2; KSS1; SSK1; SGD1; CLB2; PTP3; STE12; BEM3; HOG1; TEC1; YPD1; STE11; STE7; SLN1; PTP2; SKM1; FUS3; NBP2; FPS1; RGA1; RDI1; ARGR2; KAR4; SRL2; STE50; NDD1; SKO1; MCM1; SMP1; STE2; SSU81; ARG82; ARGR1; FLO8; PTK1; FLO1; FLO10; MATA2;	18	75
18	4	CDC42; CDC24; SWE1; BEM1; CLA4; LTE1; PEA2; SPA2; STE20; ACT1; DIG1; RGA2; SSK2; BUB2; PBS2; DIG2; KSS1; SSK1; RHO1; CDC37; STE12; BEM3; HOG1; STE4; YPD1; STE11; STE7; SLN1; SKM1; FUS3; GPA1; NBP2; RGA1; GIC1; RDI1; KAR4; MSB2; STE50; STE18; STE5; MPS1; STE2; FUS2; SSU81; RHO3; AKR1; FUS1; KIN4;	17	67
19	4	CDC42; CLA4; STE20; PRE9; BMH1; RCK2; RPN1; PRE2; PRE1; PRE10; PRE3; PRE5; PRE6; PRE7; PRE8; PUP1; PUP2; SCL1; BMH2; PRE4; PUP3; SNF1; HSP104; IRA2; RIM15; SSK2; PBS2; SSK1; RAS2; RAS1; GPA2; MSN4; YPD1; IRA1; STE11; SLN1; SKM1; SSK22; CPR7; RGA1; STB5; RDI1; SRL2; MSN2; SKN7; HSF1; SSU81; ACM1; SCH9; PTK1;	20	83
20	4	CDC42; CDC24; CLA4; STE20; RCK2; SSK2; PBS2; KSS1; SSK1; PTP3; CDC37; STE12; BEM3; HOG1; STE4; YPD1; STE11; STE7; SLN1; PTP2; SKM1; SSK22; FUS3; GPA1; NBP2; FPS1; RGA1; RDI1; MSB2; STE50; STE18; SKO1; STE5; MPS1; SKN7; SSU81; RHO3; FUS1;	24	75
average			20	77

(PPI score threshold: 0.800, credible PPI score threshold: 0.980, DFS path length: 5)
